# Circumscribed Palmar Hypokeratosis With Superimposed Actinic Keratosis

**DOI:** 10.7759/cureus.34401

**Published:** 2023-01-30

**Authors:** Erica Aukerman, Megana Rao, Azadeh Samiei, Maria C Bell, Sahand Rahnama-Moghadam

**Affiliations:** 1 Dermatology, Indiana University School of Medicine, Indianapolis, USA; 2 Pathology, Indiana University School of Medicine, Indianapolis, USA

**Keywords:** non-melanoma skin cancer, psoriasis, hand, actinic keratosis, circumscribed palmar hypokeratosis

## Abstract

A man in his late 70s with a history of psoriasis and non-melanoma skin cancer presented with a progressive rash on his right thenar eminence. He first noticed it about one year ago. He denied any pruritus in the affected region but did note some overlying skin breakdown. He had used topical betamethasone and calcipotriene cream in the past with minimal improvement. Physical examination revealed a pink atrophic plaque with linear hyperkeratotic borders and central fissuring on the right thenar eminence extending into the first webspace. A shave biopsy revealed hypokeratosis with a rim of surrounding hyperkeratosis and associated parakeratosis, basal keratinocyte atypia, and lichenoid inflammation. These histopathological features were consistent with circumscribed palmar hypokeratosis and central actinic keratosis. Circumscribed palmar hypokeratosis is often considered a benign entity, but there have been reports suggesting an association with premalignancy. The decision was made to treat with 5-fluorouracil and calcipotriene cream twice daily for six weeks. At his two-month follow-up, he endorsed a robust reaction, which was further suggestive of premalignant change. He had a near-complete resolution of the rash. This case features circumscribed palmar hypokeratosis and suggests a novel treatment option for patients who develop concomitant actinic keratosis.

## Introduction

Circumscribed palmar hypokeratosis (CPH) is characterized by a well-circumscribed, depressed, erythematous lesion on the palm. It often presents as an asymptomatic plaque with a raised scaly border. CPH has a predilection for the thenar and hypothenar eminences, but it can present elsewhere on the palm in rare cases. Middle-aged to elderly females are most commonly affected.

The first cases of CPH were reported as benign entities of unknown origin [1]. Since that time, fewer than 100 cases have been reported in the literature though it is thought to be underdiagnosed. CPH may be misdiagnosed as porokeratosis or squamous cell carcinoma in situ. The pathogenesis remains unknown, and no definitive treatment algorithm has been established. Topical steroids, calcipotriene, and 5-fluorouracil have been reported in the literature as potential treatment options with varying degrees of success. Other therapies often used in the setting of actinic damage, including cryotherapy and photodynamic therapy, may be considered depending on patient preference [2].

CPH is characterized histologically by localized thinning of the stratum corneum that is sharply distinct from the surrounding normal skin. The corneocytes in the affected region also appear more eosinophilic than those in the unaffected skin. Of note, parakeratosis and atypical keratinocytes are typically not seen, and there are no associated changes in the dermis.

## Case presentation

**Figure 1:**
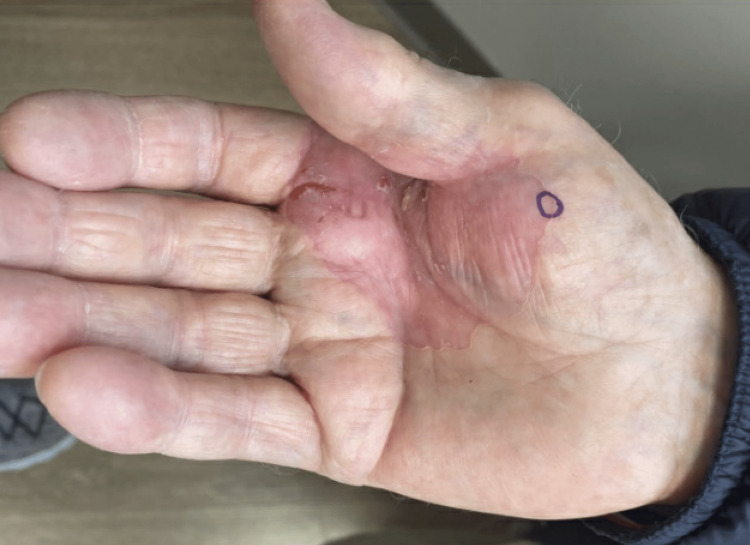
Pink atrophic plaque with linear hyperkeratotic borders, central fissuring, and erosions extending from the right thenar eminence to the first webspace

A 79-year-old male with a history of plaque psoriasis and squamous cell carcinoma presented to the clinic with concern for a rash on his right thenar eminence. He dealt with a similar rash many years ago but noticed that it resurfaced about one year ago. He described it as slowly enlarging, and he recently noticed some overlying skin breakdown. He denied any pruritus or tenderness in the affected region aside from the areas of denuded skin (Figure 1). He treated the rash with topical betamethasone ointment and calcipotriene cream for weeks with minimal-to-no improvement.

The patient’s dermatologic history is notable for a long-standing history of psoriasis that had recently been well-controlled on secukinumab. He had no associated psoriatic arthritis or nail changes. He had also previously undergone Mohs surgery for cutaneous malignancies in his right hand. In 2003, he had Mohs surgery on the right thenar eminence to treat squamous cell carcinoma in situ requiring a skin graft. He subsequently developed ulceration along the right thenar eminence due to tension in his skin graft in 2014. A repeat biopsy of the graft site at that time was negative for malignancy but consistent with psoriatic eruption. Notably, some atypia was found in the keratinocytes of this biopsy. This was successfully treated with topical corticosteroids and calcipotriene.

**Figure 2:**
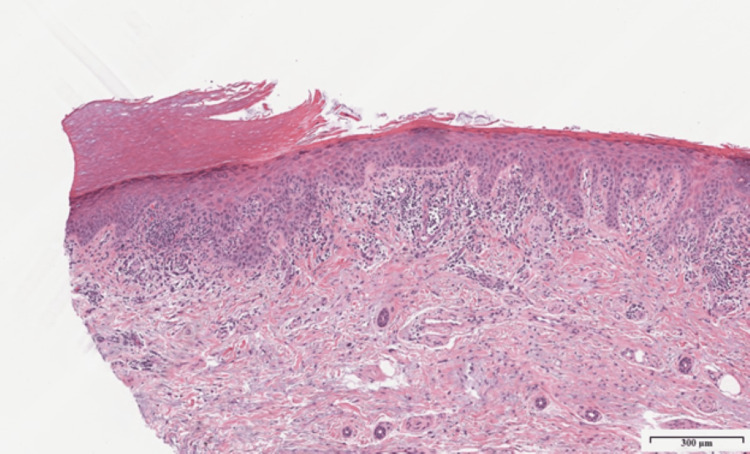
Punch biopsy demonstrating sharply demarcated hypokeratosis of stratum corneum adjacent to normal-appearing acral skin, a characteristic finding in CPH (Hematoxylin and eosin stain; original magnification X50) CPH: Circumscribed palmar hypokeratosis.

**Figure 3:**
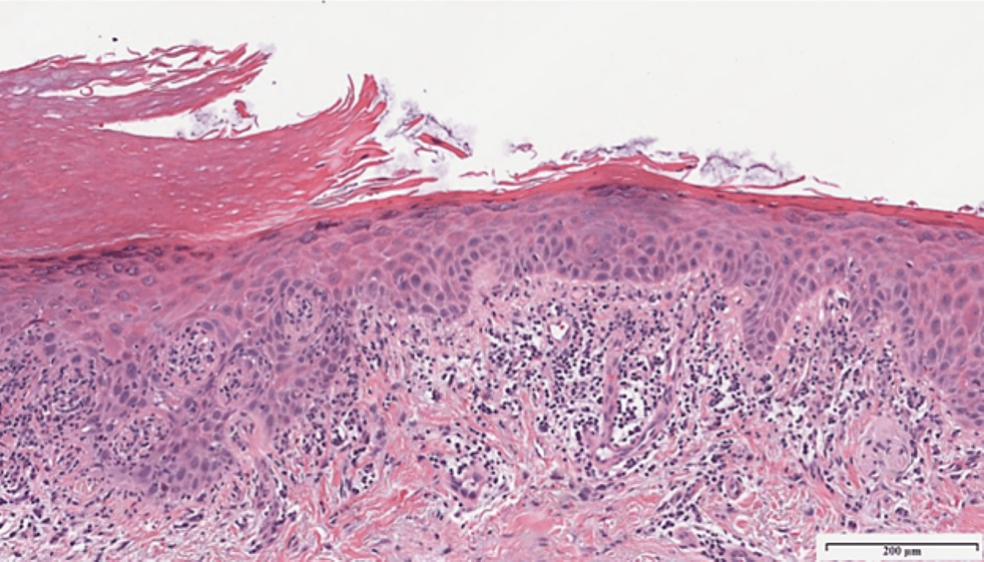
Higher power magnification demonstrating parakeratosis, basal keratinocyte atypia, and lichenoid inflammation (Hematoxylin and eosin stain; original magnification X100)

Physical examination revealed a pink atrophic plaque with linear hyperkeratotic borders along the right thenar eminence extending to the first webspace (Figure 1). Within the plaque, there were numerous fissures and shallow erosions. Based on clinical assessment, the differential diagnoses included CPH, porokeratosis, palmar pustulosis, squamous cell carcinoma, and contact dermatitis. A punch biopsy was performed to further elucidate the diagnosis. The biopsy revealed a sharply demarcated hypokeratosis of the epidermis with some parakeratosis and basal keratinocyte atypia (Figures 2, 3). There was mild to moderate solar elastosis. The acrosyringia were uninvolved by the atypical basal keratinocytes. Mild lichenoid lymphocytic inflammation was also noted. The periphery of the punch biopsy showed hyperkeratosis, a normal finding for an acral site. No underlying dyskeratotic cells were noted in the dermis. No features of psoriasis were appreciated. Parakeratosis was evident only at the hypokeratotic zone. Cornoid lamellae, which would suggest a porokeratosis variant, were not seen at the margins of the lesion. Histopathology was deemed consistent with CPH but showed early signs of basal keratinocyte atypia. The patient was prescribed a combination of 5-fluorouracil and calcipotriene cream to apply to the rash for six weeks.

**Figure 4:**
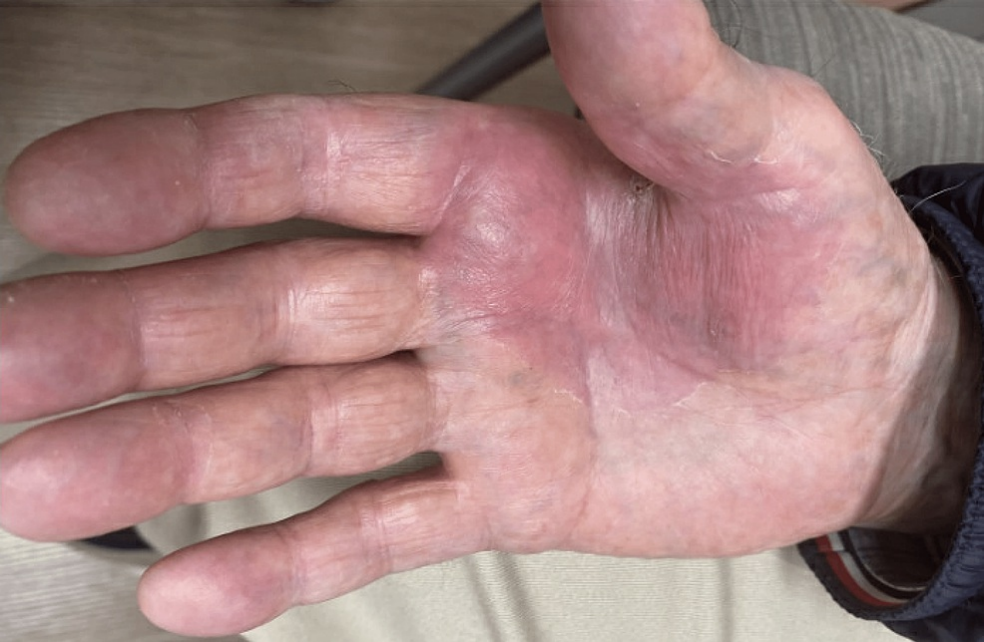
Small heme-crusted papule in the right first webspace with some faint erythema and scale and otherwise normal skin; this figure shows the improvement at two-month follow-up.

At his two-month follow-up, he reported an exuberant reaction in the affected region with significant improvement. Physical examination revealed a small heme-crusted papule at the right first webspace with some faint scale and erythema and otherwise normal skin (Figure 4). The papule at the right first webspace looked consistent with prurigo changes. The patient was prescribed triamcinolone 0.1% ointment to use on this small region, and the decision was made to hold off on further 5-fluorouracil/calcipotriene treatment. At his three-month follow-up, the rough papule in his right first webspace had completely resolved, and his right thenar eminence appeared completely healed.

## Discussion

CPH has historically been referred to as an epidermal malformation, favoring a benign nature [1]. While this condition is typically considered a benign dermatosis, there have been a few reports of associated premalignant and malignant changes [3-5]. This case demonstrates a presentation of CPH with features of basal keratinocyte atypia and lichenoid inflammatory infiltrate. The lesion was clinically concerning for malignancy, given its history of increasing size and occurrence in a site of previously excised squamous cell carcinoma. While typical features of CPH were seen, less commonly reported premalignant traits of basal keratinocyte atypia, lichenoid inflammatory infiltrate, and parakeratosis at the zone of hypokeratosis were also present. This patient had a robust response to topical 5-fluorouracil, further supporting malignant potential.

As mentioned previously, the etiology and pathogenesis behind CPH are largely unknown; however, there are several hypotheses, including response to trauma, human papillomavirus (HPV), keratinization disturbances, and epidermal hyperproliferation associated with keratinocyte fragility [6]. More recently, there was a report of a patient who developed actinic keratosis within an area of hypokeratosis [7]. The authors proposed that shedding of the stratum corneum may trigger hyperproliferation of the epidermis in an attempt to replace the stratum corneum. Thinning of the stratum corneum may also make keratinocytes more vulnerable to ultraviolet damage. Additionally, irritation or microtrauma that occurs in the area can cause further desquamation [8]. Our patient exhibited multiple biopsy-proven lichenoid hypertrophic actinic keratoses (AKs) near the right thenar eminence, leading to localized chronic irritation. It is possible that our patient developed CPH as a result of chronic irritation and microtrauma secondary to AKs and procedures. He may also be at risk of developing further premalignant or malignant lesions within the area of CPH as a result of a thin stratum corneum and the two proposed consequences (reflex epidermal hyperproliferation and exposed keratinocytes).

## Conclusions

This report demonstrates a case of CPH with histologic superimposed actinic keratosis responding to a combination of topical 5-fluorouracil and calcipotriene therapy. While CPH is often considered benign, treatment should be initiated in cases where keratinocyte atypia is present even if the lesion is not bothersome to the patient. In addition, asymptomatic lesions with benign pathology should be followed regularly for signs of malignant transformation.
